# Prediction of mortality in metastatic colorectal cancer in a real-life population: a multicenter explorative analysis

**DOI:** 10.1186/s12885-020-07656-w

**Published:** 2020-11-25

**Authors:** Holger Rumpold, Dora Niedersüß-Beke, Cordula Heiler, David Falch, Helwig Valenting Wundsam, Sigrid Metz-Gercek, Gudrun Piringer, Josef Thaler

**Affiliations:** 1 Gastrointestinal Cancer Center, Ordensklinikum Linz, Seilerstaette 4, 4010 Linz, Austria; 2 Department of Internal Medicine I, Wilhelminenspital, Vienna, Austria; 3 Department of Surgery, Ordensklinikum Linz, Linz, Austria; 4 Clinical Cancer Centre Upper Austria, Linz, Austria; 5 Department of Internal Medicine IV, Hospital Wels-Grieskirchen, Wels, Austria

**Keywords:** Nomogram, Mortality, Metastatic colorectal cancer, Real-life, Prognosis

## Abstract

**Background:**

Metastatic colorectal cancer (mCRC) remains a lethal disease. Survival, however, is increasing due to a growing number of treatment options. Yet due to the number of prognostic factors and their interactions, prediction of mortality is difficult. The aim of this study is to provide a clinical model supporting prognostication of mCRC mortality in daily practice.

**Methods:**

Data from 1104 patients with mCRC in three prospective cancer datasets were used to construct and validate Cox models. Input factors for stepwise backward method variable selection were sex, *RAS/BRAF*-status, microsatellite status, treatment type (no treatment, systemic treatment with or without resection of metastasis), tumor load, location of primary tumor, metastatic patterns and synchronous or metachronous disease. The final prognostic model for prediction of survival at two and 3 years was validated via bootstrapping to obtain calibration and discrimination C-indices and dynamic time dependent AUC.

**Results:**

Age, sidedness, number of organs with metastases, lung as only site of metastasis, *BRAF* mutation status and treatment type were selected for the model. Treatment type had the most prominent influence on survival (resection of metastasis HR 0.26, CI 0.21–0.32; any treatment vs no treatment HR 0.31, CI 0.21–0.32), followed by *BRAF* mutational status (HR 2.58, CI 1.19–1.59). Validation showed high accuracy with C-indices of 72.2 and 71.4%, and dynamic time dependent AUC’s of 76.7 ± 1.53% (both at 2 or 3 years), respectively.

**Conclusion:**

The mCRC mortality prediction model is well calibrated and internally valid. It has the potential to support both, clinical prognostication for treatment decisions and patient communication.

**Supplementary Information:**

The online version contains supplementary material available at 10.1186/s12885-020-07656-w.

## Background

Colorectal cancer (CRC) is one of the most common malignant diseases in the world and has also one of the highest cancer-related mortality rates [1, 2]. Fortunately, both incidence and mortality from CRC have decreased over the last decades. This is due to several factors, but the most important ones are successful screening and new systemic, as well as progressive surgical treatment options [3]. Nonetheless, metastatic CRC (mCRC) remains a lethal disease that is present in about 20–25% of patients at diagnosis and 30% will experience a metastatic relapse after initial curative surgical treatment, with or without adjuvant chemotherapy [4]. In mCRC, estimating survival is difficult, even for experienced oncologists. This directly influences the quality of the communication with patients as information regarding an accurate prognosis is one of the most important pieces of information provided to patients by oncologists [5]. Sharing information about the course of the disease with patients and estimating survival are challenging issues as is evident by the numerous prognostic and/or predictive factors that have been described over recent years. Socioeconomic factors, supportive treatment enabling adherence to planned treatment procedures, exercise programs and patient reported outcomes play a significant role in prognostication [6]. Other factors of relevance in clinical practice include the tumor load measured by the number of organs involved in metastasis [7, 8], patterns of metastatic spread [9], primary tumor location [10–12] and metachronous or synchronous disease [13]. Currently, additional molecular markers and even molecular signatures have enriched the traditional family of markers, adding significantly to the complexity of mortality prognosis [14]. It is now established that *BRAF* mutated patients show the worst prognosis among all patients [15] and the consensus molecular subtypes give a deeper insight into the biology of the disease [16, 17]. Along with the advent of these modern markers, systemic treatment options targeting angiogenesis, the epidermal growth factor pathway (EGFR) or the *BRAF*-pathway have been developed that enable the use of individual treatments [18]. For example, metastastic left sided colorectal cancer that is *RAS* wild-type and treated by an EGFR-antibody combined with chemotherapy shows the longest survival [19]. In addition, patients who are *BRAF* mutated benefit from targeted treatment rather than from chemotherapy [20].

These improvements in systemic treatments have been accompanied by progressive surgical treatment concepts, which include the resection of metastases, even repeatedly and in more than one organ. For such multimodal treatment concepts, patient selection is the key to success. A multidisciplinarity approach encompassing the experience of specialized oncologists, surgeons and radiologists has become a hallmark of optimal clinical outcomes in the treatment of mCRC resulting in overall survival rates of more than 5 years in the best cases [21]. The complex prognostic interplay between anatomic, pathological, molecular, and clinical factors cannot be captured in clinical trials. Tools allowing a more accurate prognostication, however, are highly desirable. These are not only helpful in clinical practice, but also support the design of new trials and the evaluation of novel treatments that may play a part in improving routine care. There have been a few contributions to date to fill this gap. Two research groups reported a model tested in large patient populations, however they only include patients treated within clinical trials since 1997, thereby not ideally representing a real-life population of colorectal cancer patients treated with modern, multidisciplinary treatments [22, 23]. Mortality determinants were also studied in a real-life cohort treated before 2010 and reported single factors associated with a higher relative risk of early mortality, yet representing a patient population before the era of modern oncology [24, 25]. Ge and colleagues described a nomogram for mCRC patients based on parameters derived from the Surveillance, Epidemiology, and End Results Program (SEER)-database; they identified 13 factors associated with survival at three and 5 years but only treated patients were included and the remarkably high number of prognostic factors limit its clinical utility [26]. Additionally, the vast majority of patients suffering from mCRC will survive approximately two to 3 years, which has been shown for patients treated either in clinical trials [27, 28] or in a real-life setting [9, 29]. To our knowledge, a prognostic model considering this particular survival time in a real-life patient cohort treated by contemporary treatments has not yet been reported.

We therefore aim to validate a mortality prediction model in a representative real-world patient cohort with mCRC by identifying factors directly associated with patient survival at 2 and 3 years. By providing this work, we hope to support oncologists to communicate individualized survival estimation to every patient in daily practice without incurring additional cost.

## Methods

### Patient sample

**Fig. 1:**
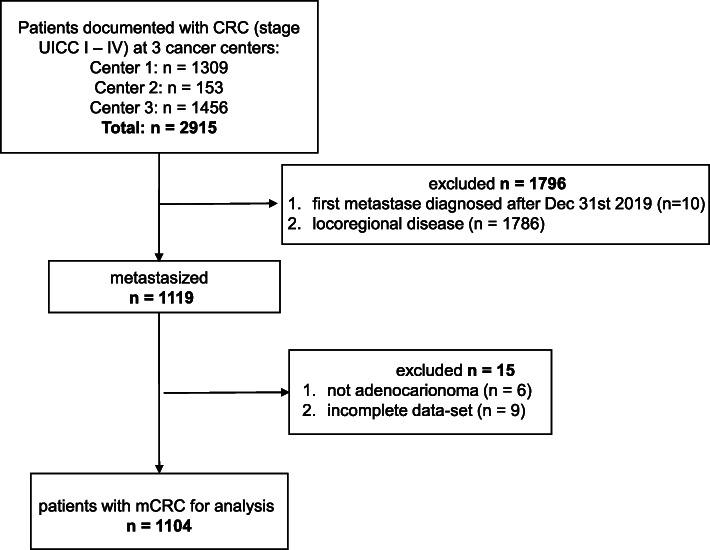
Flow chart describing patient selection for inclusion into the analysis

We retrospectively identified 2915 patients diagnosed with colorectal cancer treated at three different oncological centers from January 2006 to March 2020 in Austria. Clinical data were obtained either from a monitored cancer registry (two centers) or by chart review (one center). Patients with metastasized colorectal carcinoma who developed metastases before Dec 31th 2019 were included in the analysis. Further, only adenocarcinoma of the colon or rectum were included and patients without complete follow up data were excluded (Fig. 1). Treatment decisions were based on local guidelines including contemporary systemic treatment with or without resection of metastasis.

### Survival analysis and nomogram development

The endpoint of this study was overall survival (OS) defined as the time between first diagnosis of metastatic disease by histology and death. Kaplan-Meier-estimates and curves were used as descriptive measures for survival data. The effects of predictors on overall survival were investigated separately with Kaplan-Meier curves. The reverse Kaplan-Meier method was used for calculation of median follow-up time. A non-normal distribution of continuous variables was assumed and medians with interquartile ranges (IQR) were reported. Correlation coefficients, Cramers V, point-biserial correlation and eta-squared values were used to describe eventual pairwise dependencies between potential predictive parameters. A stepwise backward method according to the Akaike Information Criterion (AIC) was used for automatic variable selection [30]. Cox regression models were used to analyze the effects of predictors in multifactorial models. The following assumptions of Cox regression models were checked: (i) proportionality of hazards by inspecting the Kaplan-Meier curves and by graphically checking independency of residuals over time; (ii) linearity of continuous predictors by plotting marginal residuals with and without different transformations; and (iii) outliers and influential cases were deleted when dfbeta-values were higher than 2/sqrt(N) = 0.06 [31]. Additionally, deviance from symmetry was checked graphically. A nomogram based on the final model was constructed for the probability of survival at 24 and 36 months.

### Performance of the nomogram

Validation of the nomogram was done using a bootstrap strategy of 2000 resamples as utilized by similar research [22, 23, 26, 32, 33]. The discrimination ability of the nomogram was evaluated by calculating the concordance index (C-Index). This index should be greater than 50% (100% in a perfect model) [34]. The nomogram was calibrated by bootstrapping (k = 2000) of five groups with differing survival probabilities; predicted survival probability was plotted against the observed survival. The accuracy of the nomogram was quantified and compared using the time dependent AUC-values.

For all analyses the R version 3.6.3 (R core Team, 2020) was used with the packages scoredardModelUtils (pairwise Cramers V), lsr (eta-squared), survival (survival analyses, Kaplan-Meier-analyses, cox-regression, diagnostics), survminer (survival-curves, diagnostics), pec (C-index), rms (calibration, validation, nomogram), timeROC (time dependent AUC-values). A significance level of 5% was assumed in all analyses.

## Results

### Characteristics of the patient sample

In total, 2915 patients diagnosed with colorectal cancer were identified in three distinct registries. Patients who did not have metastatic disease by Dec 31st 2019 (*n* = 1796) were excluded from the analysis. Thus, a total of 1119 patients were either synchronously or metachronously metastasized and therefore eligible for inclusion. From this sample 15 patients were excluded due to a histology type other than adenocarcinoma or a lack of sufficient data for survival time calculation. This resulted in a total sample of 1104 patients (Fig. 1). Median age was 67.4 years; most patients had synchronous disease defined as diagnosis of metastasis within the first 6 months after diagnosis of the primary tumor. Molecular markers were not consistently available. When separating at the left flexure, 70% showed a left sided colon or rectum carcinoma and 30% a right sided colon cancer. The majority of patients showed metastases in one organ only at time of diagnosis, which predominantly was the liver (Table 1). The median OS of the whole cohort was 20 months (CI 18.5–22.3). The treatment dependent OS was 6.27 (CI 4.11–7.82) months for patients receiving no treatment at all (best supportive care; BSC) and 26.61 months (CI 24.05–28.45) for patients receiving any treatment, defined as either systemic treatment alone, metastasis resection alone (predominantly in case of metachronous, oligometastatic relapse) or multimodal treatment including metastasis resection and systemic treatment. The resection rate of metastases was 20.7% in the whole population and a proportion of patients (24.8%) received BSC. A total of 67.7% (746 patients) of patients received at least one line of systemic antineoplastic treatment and of these, 62.3% (465 patients) received at least 1 second and 35% (261 patients) at least one third line of treatment.

**Table 1:** Demographics of patients

Patients, n	1104
Age (median [IQR])	69.00 [60.00, 76.00]
Sex	
female	410 (37.1)
male	694 (62.9)
Stage at diagnosis	
UICC I	34 (3.1)
UICC II	91 (8.2)
UICC III	263 (23.8)
UICC IV	716 (64.9)
Time to metastasis	
DFS < 6 mts	771 (69.8)
DFS > 6 mts	333 (30.2)
Treatment modality	
best supportive care	274 (24.8)
resection of metastases only	84 (7.6)
systemic treatment and resection of metastases	144 (13.0)
systemic treatment only	602 (54.5)
Treatment lines	
first line treatment	746 (67.6)
second line treatment	465 (42.1)
third line treatment	261 (23.6)
RAS-status	
RAS-mutated	453 (41.0)
RAS-wildtype	426 (38.6)
unknown	225 (20.4)
BRAF-status (%)	
BRAF-mutated	42 (3.8)
BRAF-wildtype	364 (33.0)
unknown	698 (63.2)
MMR-status	
microsatellite instable	26 (2.4)
microsatellite stable	180 (16.3)
unknown	898 (81.3)
Location of primary	
colon	699 (64.2)
rectum	389 (35.8)
LCC	762 (70.0)
RCC	326 (30.0)
Organs with metastases	
1 organ	784 (71.0)
2 or more organs	320 (29.0)
Lung only metastases	
lung only	111 (10.1)
other than lung only	993 (89.9)

Values are given in n (%) unless not otherwise described; DFS disease free survivial; mts months

### Prognostic model

**Fig. 2:**
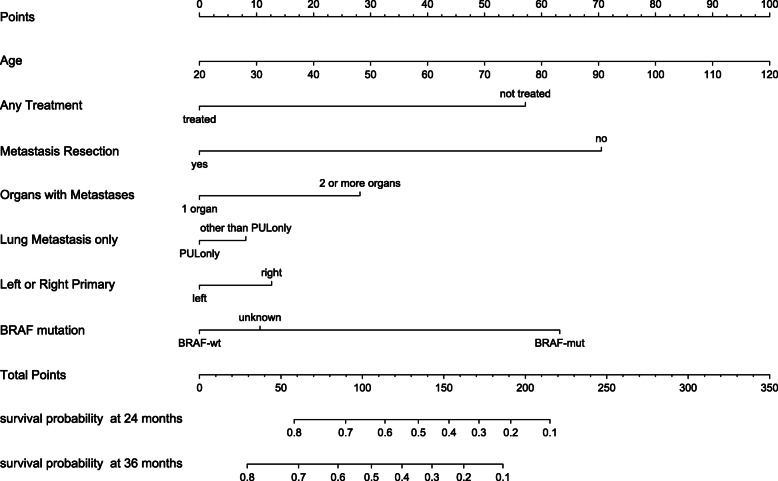
Nomogram allowing prediction survival at 24 and 36 months

In 1104 patients the following parameters were analyzed for statistical significance in a stepwise backward method according to AIC: disease free survival (DFS), rectum or colon, left (LCC) or right (RCC) colon cancer, mutation status in *BRAF* and *RAS*, microsatellite status, age, gender, constellation of metastases (single organ, multiple organs), metastatic patterns (detailed information on which organ/organs were affected by metastasis), treatment modality (no treatment, systemic treatment only, multimodal treatment including systemic treatment and metastasis resection or metastasis resection alone if applicable) and receiving systemic treatment with or without monoclonal antibodies. These parameters were selected, because significant correlation with each other was not present and each of it showed significant impact in univariate analysis (data not shown). Treatment type was found to be the most relevant prognostic parameter (HR 0.31, CI 0.26–0.36), especially if a resection of metastases was performed (HR 0.26, HR 0.21–0.32). The presence of a lung metastasis at diagnosis only was of positive prognostic value (HR 0.68, CI 0.53–0.87). A worse mortality outcome was found for having more than two organs affected by metastasis (HR 1.5, CI 1.3–1.74) and patients with RCC fared worse than those with RCC (HR 1.37, CI 1.19–1.58); *BRAF* mutated tumors showed worst survival (HR 2.58, CI 1.67–3.99). All variables showed statistically significant HR’s (Table 2). The selected parameters were weighted against each other in a nomogram, which finally allows the prediction of survival at 24 or 36 months, respectively (Fig. 2).

**Table 2:** HR of prognostic factors

	HR	CI	*p*
RCC vs LCC	1.37	1.19–1.58	< 0.001
BRAF mutated vs wildtype	2.58	1.67–3.99	< 0.001
Resection of metastasis vs not	0.26	0.21–0.32	< 0.001
Treated vs not	0.31	0.26–0.36	< 0.001
Age > 60 vs below	1.61	1.37–1.89	< 0.001
2 or more organs with metastases	1.5	1.3–1.74	< 0.001
Lung metastasis only vs not	0.68	0.53–0.87	0.002

*HR* Hazard ratio, *CI* Confidence intervall, *RCC* right sided colon cancer; *LCC* left sided colorectal cancer

### Validation and calibration of the prognostic model

Accuracy of the nomogram showed strong internal validity by a time dependent AUC of 76.7 ± 1.53% at 24 months and 77.9 ± 1.72% at 36 months. This was confirmed by the additional calculation of the C-Index, which resulted in a discrimination of 72.2% at 24 months and 71.4% at 36 months (Supplementary Figure 1).

**Fig. 3:**
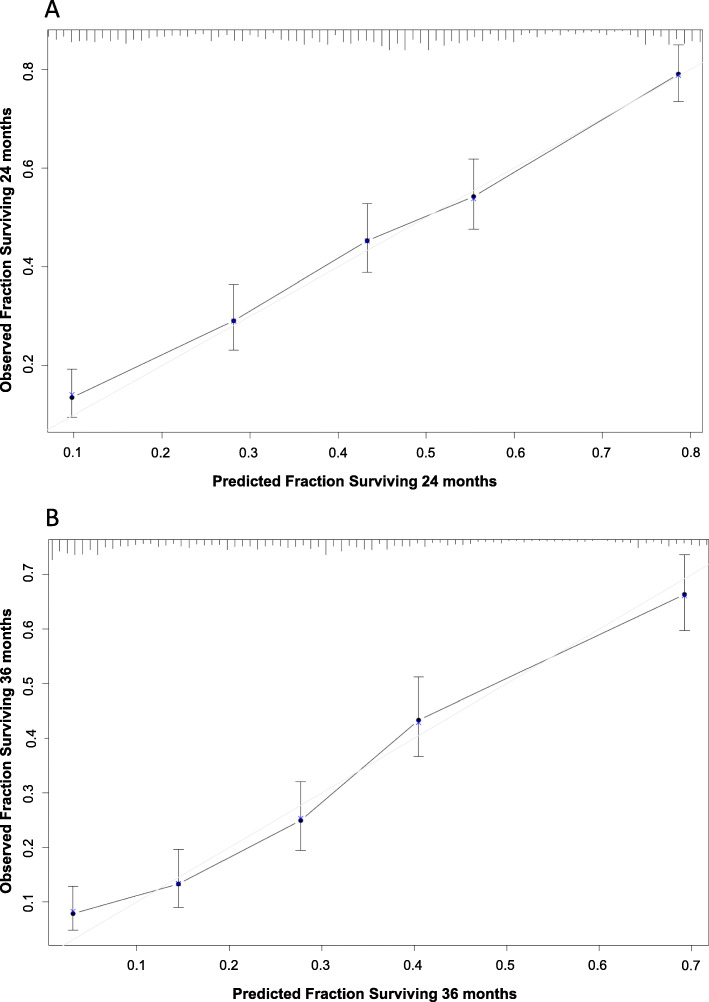
Calibration of the nomogram at 24 months (**a**) and 36 months (**b**)

The performance of our model was calibrated by bootstrapping (k = 2000) of five groups, consisting of 215 patients each, with differing survival probabilities. This calculation showed a reliable concordance between predicted and observed survival in the cohort at 24 months and 36 months (Fig. 3).

## Discussion

In the presented study a nomogram predicting survival at 24 and 36 months for patients suffering from mCRC was developed and validated. The model is based on the clinical features of 1104 patients and also includes a differentiated view of modern treatment types. The performance of the model in terms of discrimination, calibration and clinical utility is high and reliable.

The selection of variables was based on easily obtained clinical parameters and on treatment types that could potentially be applied to a patient. To our knowledge, this question has not been addressed in this detail in a real-life population treated by contemporary treatment concepts so far. Of the many factors known to be of prognostic relevance, age, number of organs affected by metastasis, lung metastasis only, left or right primary, *BRAF* mutational status and especially treatment modality were selected for the model after a stepwise exclusion in a multivariate cox-regression model. Age as one of the relevant prognostic factors has also been reported to be associated with the patients’ survival in several other tumor entities [35, 36]. But although it has been reported, the specific mechanisms remain unclear. Chronic inflammation or lower immune responses have been discussed as playing an important role [37, 38]. In our study the survival of patients older than 60 years was worse compared to patients less than 60 years old. This might be due to the higher rate of BSC and less intense treatment regimen found in older patients (data not shown). This correlation has also described in a similar fashion by others [26, 39].

The number of organs involved in metastatic disease and tumor load [40], the pattern of metastasis [9, 41], left or right primary location [11] and *BRAF* mutational status [42] are known and well described prognostic factors in this population. Among these, in our sample *BRAF* appeared to have a major impact on survival despite the relatively low number of patients. Furthermore, treatment type was found to have the strongest effect, resulting in the exclusion of known prognostic factors. One of these, for example, was DFS, which potentially discriminates patients with good prognosis from patients with bad prognosis (synchronous vs. metachronous disease; data not shown). We differentiated between patients receiving either best supportive care, systemic treatment only, multimodal treatment including systemic treatment and resection of metastasis or resection of metastasis only. The main reason for best supportive care were comorbidities, which implies a higher risk than benefit from treatment. A considerable fraction of approximately 25% of patients were allocated to this category. Second line treatment was given to 62% of the patients and 35% of patients received a third line therapy. These frequencies are comparable to an analysis of subsequent treatments in the FIRE-3 trial population, where 70 and 43% received second and third line treatment, respectively [43]. The slightly lower rates in our analysis can be explained by obvious differences between our real world cohort and a study population that included selected patients only. Consideration of treatment type in this detail is of high importance as especially multimodal treatment concepts have been proven to be of high efficacy if performed in a multidisciplinary setting [21]. The nomogram shows the weighted impact of the factors on all-cause mortality at 24 and 36 months. The 13 factors associated with survival at 3 and 5 years identified by Ge and colleagues [26] is a fairly high number of variables for use in daily practice and as the median OS in mCRC is found to be around 30 months, the time points allow the prediction of long term survival only. This might have been intentional since the study had only one focus on resection of metastases. Such patients are known for survival times that are beyond the usual median OS. We intended to cover the majority of patients and therefore describe survival at 24 and 36 months, which is fairly around the median OS described in the literature. Most studies investigating the interplay of prognostic relevant factors are limited to patients that received at least one treatment [26] or were even treated within clinical trials [22, 23]. Both are not optimally representative of a real-world scenario. Having this in mind, we also included patients who were considered to be unfit for treatment. This provides information on a representative spectrum of patients, of interest for everyday practice.

When comparing the performance of our models, the most suitable trial is by Ge et al. who reported a C-Index of 0.69 [26]. Another study reporting on a model for prediction of disease free and overall survival after first line treatment with a chemotherapy doublet showed a C-Index of 0.66 [33]. The C-Index of our model was 72% at 24 months and 71% at 36 months, implying an accuracy comparable to these publications. However, for t-year predictions the dynamic time-dependent AUC (dtdAUC) is reported as being more reliable [44]. In our model the tdAUC at 24 months was 77 and 78% for 36 months. These values indicate a high accuracy of the nomogram.

Several potential limitations should be considered when interpreting the results of our study. Foremost, it is a retrospective study, which implies the risk of a selection bias. Unfortunately, the rate of unknown mutation status of *RAS* and *BRAF*, as well as information on microsatellite status was high, which may have led to an underestimation of the influence of *RAS* mutation status and treatment with EGFR antibodies on survival. A prospective evaluation of the presented model and its applicability in daily practice is needed.

## Conclusion

In conclusion, we present a clinical model that supports the prediction of the most relevant 2- and 3-year survival in mCRC, focussing on clinicopathological features and on different treatment types covering all conceivable treatment concepts in the modern oncological treatment era. As the patients included in our analysis were treated at three different centers our patient cohort may be considered representative of a real-world scenario. This predictive mortality model may contribute to the difficult but important clinical issue of prognostication in patients with mCRC by supporting communication to the patients and the decisions on treatment strategy.

## Supplementary Information

**Additional file 1.**

## Data Availability

The datasets used and/or analysed during the current study are available from the corresponding author on reasonable request.

## Abbreviations

CRC: Colorectal cancer
mCRC: Metastatic colorectal cancer
BRAF: B-raf (rapidly accelerated fibrosarcoma), proto-oncogene
EGFR: Epidermal growth factor receptor
RAS: Ras (rat sarcoma), proto-oncogene
SEER: Surveillance, epidemiology, and end fesults program
IQR: Interquartile ranges
AIC: Akaike information criterion
CI: Confidence interval
HR: Hazard ratio
C-Index: Concordance index
AUC: Area under the curve
tdROC: Time dpendent AUC-values
BSC: Best supportive care
DFS: Disease free survival
LCC: Left sided colon cancer
RCC: Right sided colorectal cancer
OS: Overall survival
