# Supplementary figures and images for: Prediction of mortality in metastatic colorectal cancer in a real-life population: a multicenter explorative analysis
Source: BMC Cancer. 2020 Nov 25;20:1149. doi: 10.1186/s12885-020-07656-w (PMC7691098; doi:10.1186/s12885-020-07656-w)

**A**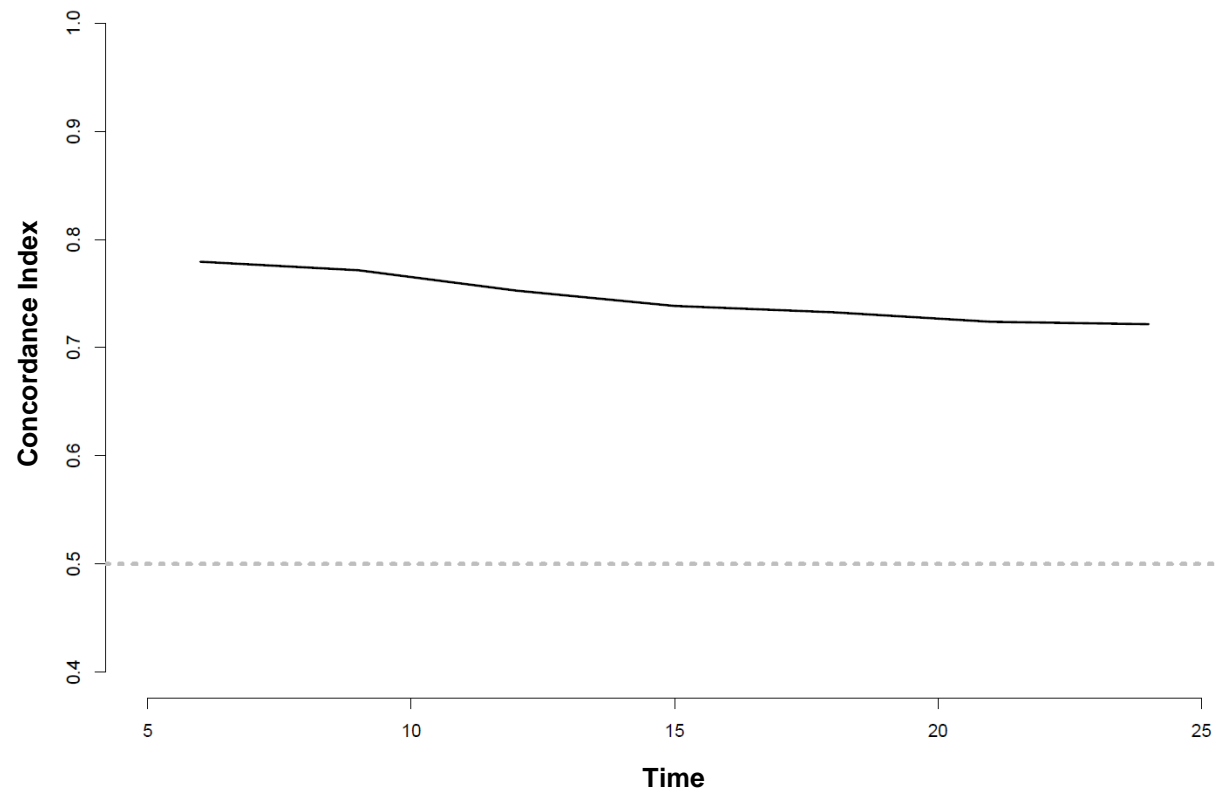**B**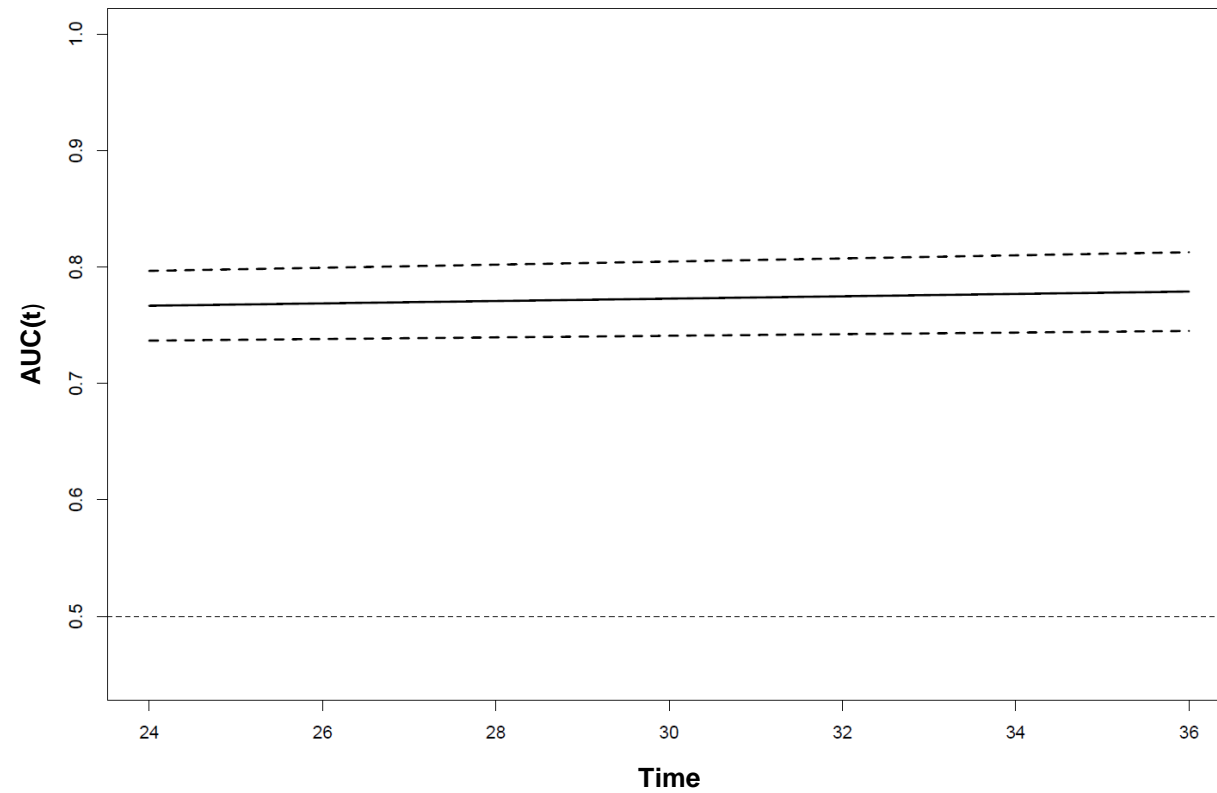

Supplement: Supplementary file 1 — Additional file 1. [file 12885_2020_7656_MOESM1_ESM.pdf]
